# A Review of Fibrocartilaginous Embolic Myelopathy and Different Types of Peracute Non-Compressive Intervertebral Disk Extrusions in Dogs and Cats

**DOI:** 10.3389/fvets.2015.00024

**Published:** 2015-08-18

**Authors:** Luisa De Risio

**Affiliations:** ^1^Centre for Small Animal Studies, Animal Health Trust, Newmarket, Suffolk, UK

**Keywords:** dog, cat, fibrocartilaginous embolic myelopathy, non-compressive nucleus pulposus extrusion, intramedullary intervertebral disk extrusion

## Abstract

This review discusses terminology, pathological, clinical, and magnetic resonance imaging (MRI) findings, treatment, outcome, and prognostic factors of fibrocartilaginous embolic myelopathy (FCEM), acute non-compressive nucleus pulposus extrusion (ANNPE), and intradural/intramedullary intervertebral disk extrusion (IIVDE). FCEM, ANNPE, and IIVDE have a similar clinical presentation characterized by peracute onset of neurological dysfunction that is generally non-progressive after the initial 24–48 h. Differentiating between these conditions can be challenging, however, certain clinical and imaging findings can help. FCEM can occur in both adult and immature animals, whereas ANNPE or IIVDE have been reported only in animals older than 1 year. In dogs, ANNPE and IIVDE most commonly occur in the intervertebral disk spaces between T12 and L2, whereas FCEM has not such site predilection. In cats, FCEM occurs more frequently in the cervical spinal cord than in other locations. Data on cats with ANNPE and IIVDE are limited. Optimal MRI definition and experience in neuroimaging can help identify the findings that allow differentiation between FCEM, ANNPE, and IIVDE. In animals with ANNPE and IIVDE, the affected intervertebral disk space is often narrowed and the focal area of intramedullary hyperintensity on T2-weighted images is located above the affected intervertebral disk space. In dogs with ANNPE signal changes associated with the extruded nucleus pulposus and epidural fat disruption can be identified in the epidural space dorsal to the affected intervertebral disk. Identification of a linear tract (predominantly hyperintense on T2-weighted images, iso to hypointense on T1-weighted images and hypointense on T2*-weighted gradient recall echo images) extending from the intervertebral disk into the spinal cord parenchyma is highly suggestive of IIVDE. Treatment of FCEM and ANNPE is conservative. Dogs reported with IIVDE have been managed either conservatively or surgically. Prognostic factors include degree of neurological dysfunction (particularly loss of nociception) and disease-specific MRI variables.

## Introduction

Fibrocartilaginous embolic myelopathy (FCEM) and peracute intervertebral disk extrusions (IVDEs) that are not resulting in extradural spinal cord compression have been increasingly recognized since the use of magnetic resonance imaging (MRI) has become widespread in veterinary neurology. Studies and case reports have been published on the clinical and diagnostic features of these conditions and a few studies have also investigated outcome and prognostic factors (1–37). However, no previous comparative review has discussed similarities and differences in clinical presentations, imaging and pathological findings, outcomes, and prognostic factors of these conditions.

Terminology to describe peracute IVDEs that are not resulting in extradural spinal cord compression has sometimes been confusing in the veterinary literature as different terms have been used to refer to the same condition [e.g., acute non-compressive nucleus pulposus extrusion (ANNPE), intervertebral disk “explosion,” high-velocity–low volume disk extrusion, exercise-associated peracute thoracolumbar disk extrusion], whereas other times the same term (e.g., traumatic disk herniation) has been used to refer to both compressive and non-compressive myelopathies resulting from extrusion of either hydrated or degenerated intervertebral disk (14–20). In this review, the term peracute non-compressive IVDEs will be used to indicate IVDEs that do not result in extradural spinal cord compression.

Fibrocartilaginous embolic myelopathy and peracute non-compressive IVDEs can generally be easily differentiated from other myelopathies (such as IVDE resulting in extradural spinal cord compression, vertebral fracture/luxation, infectious/inflammatory meningomyelitis, and neoplasia) based on clinical and diagnostic investigation findings (12, 13). However, differentiation between FCEM and peracute non-compressive IVDEs can be challenging. Definitive diagnosis can be reached post-mortem. Definitive diagnosis has also been reached following surgery in some dogs with intradural/intramedullary intervertebral disk extrusions (IIVDEs) (23, 24, 26–28, 30, 31, 33–37). Ante mortem diagnosis is largely supported by MRI. MRI characteristics of FCEM and disk-associated non-compressive myelopathies have been described predominantly in studies using high-field MRI (4, 6, 17, 18, 29). No studies have compared diagnostic accuracy for differentiating FCEM and disk associated non-compressive myelopathies using high- and low-field MRI. However, following publication of the studies on high-field MRI characteristics of FCEM and ANNPE (4, 6, 17, 18), there have been anectodal reports about the challenge in making this differentiation with low-field MRI, suboptimal MR image definition and limited experience in neuroimaging.

This review article will discuss terminology, macroscopic and histopathological findings, clinical and imaging findings that can help differentiating between FCEM and peracute non-compressive IVDEs, as well as discuss treatment, outcomes, and prognostic factors of these conditions.

## Terminology

### Fibrocartilaginous embolic myelopathy

Fibrocartilaginous embolic myelopathy is a vascular disease of the spinal cord caused by embolization of spinal vasculature with fibrocartilaginous material histologically and histochemically identical to the nucleus pulposus of the intervertebral disk, resulting in ischemic necrosis of dependent regions of spinal cord parenchyma (1). The nucleus pulposus of the intervertebral disk is considered as the source of the fibrocartilaginous material. The vertebral growth-plate cartilage in immature animals and the metaplasia of the vascular endothelium have also been proposed as possible sources of the fibrocartilaginous material (2, 12, 13). Various theories have been hypothesized to explain how the fibrocartilaginous material can enter the spinal vasculature (2, 12). FCEM is considered as the most common cause of ischemic myelopathy in dogs (12).

### Peracute non-compressive intervertebral disc extrusions

The intervertebral disk can extrude following degeneration (38, 39) as well as secondary to supra-physiological forces (e.g., during spinal trauma or vigorous exercise) applied to a normally hydrated disk (14, 18). The intervertebral disk material can extrude either in an extradural, intradural-extramedullary, or intramedullary location. The extruded intervertebral disk material can result in spinal cord contusion with or without compression and rarely in laceration of the meninges with or without penetration of the spinal cord parenchyma. Terminology used in the veterinary literature to indicate extrusion of degenerated or non-degenerated intervertebral disk material, resulting in non-compressive myelopathy is discussed in the following section.

Acute non-compressive nucleus pulposus extrusion refers to the extrusion of hydrated nucleus pulposus due to a sudden increase in intradiscal pressure during vigorous exercise (such as running and jumping) or trauma (18). The hydrated nucleus pulposus herniates through a tear in the annulus fibrosus contuses the spinal cord and then dissipates within the epidural space causing minimal to no spinal cord compression. This condition has also been named as traumatic disk extrusion, traumatic disk prolapse, dorsolateral intervertebral disk “explosion,” high-velocity–low volume disk extrusion, exercise-associated peracute thoracolumbar disk extrusion, and Hansen type III intervertebral disk disease (14–17, 19). The terminology “Hansen type III intervertebral disk disease” or “type III intervertebral disk extrusion” should not be used to indicate extrusion of hydrated nucleus pulposus, resulting in non-compressive myelopathy (39). Hansen described only two types of intervertebral disk degeneration, resulting in type I and type II intervertebral disk herniation, respectively (38, 39). Type III IVDEs were originally described by Funquist as a subtype of Hansen type I intervertebral disk degeneration and herniation characterized by extension of disk material “like a carpet over several vertebrae” (40).

In this review article, extrusion of hydrated intervertebral disk material in an intradural-extramedullary or in an intramedullary location will be referred to as IIVDE.

Extrusion of hydrated nucleus pulposus can also result in various degrees of compressive myelopathy (41–43). This type of IVDE will not be discussed in this review as the extradural spinal cord compression can be easily identified on MRI performed within days of onset of myelopathy. However, follow-up MRI performed two or more months after onset has revealed complete disappearance of the extruded hydrated nucleus pulposus in conservatively treated dogs (43).

The term traumatic IVDE has been used to refer not only ANNPE but also extrusion of degenerated intervertebral disk material, resulting in contusive and compressive myelopathy following trauma to the spinal region (20). In a study including 50 dogs with a history of trauma to the spinal region, 31 (62%) had an IVDE without any other detectable vertebral lesions (20). The IVDE resulted in compressive myelopathy (by extruded disk material with or without hemorrhage) in 9 (29%) dogs and non-compressive myelopathy in 22 (71%) dogs (20).

Various descriptive terms have been used to indicate peracute extrusion of hydrated or degenerated intervertebral disk material generally during trauma or exercise, resulting in penetration of the dura mater and frequently also of the spinal cord parenchyma (23–37). In this review article, the extrusion of either hydrated or degenerated intervertebral disk material in an intradural-extramedullary or in an intramedullary location will both be referred to as IIVDE. IIVDE is considered a rare type of intervertebral disk herniation and the largest published study in the veterinary literature includes eight dogs only (37). In this recent study, the prevalence of thoracolumbar IIVDE among the total population of dogs that developed a thoracolumbar intervertebral disk herniation and that underwent spinal surgery was 0.5% (37).

## Macroscopic and Histopathological Findings

Histopathological examination allows definitive diagnosis of FCEM, ANNPE, and IIVDE and enables to ascertain whether the intervertebral disk material is degenerated or not.

### Fibrocartilaginous embolic myelopathy

Macroscopically, the affected spinal cord segment can appear normal or swollen with or without visible malacic and hemorrhagic areas depending on the severity and extent of the ischemic injury. Transverse sectioning of the affected spinal cord reveals malacia with or without concurrent hemorrhage. Histological examination of the affected spinal cord segments reveals fibrocartilaginous material in spinal vessels (arteries and/or veins) within or near an area of focal myelomalacia. The distribution of the lesion reflects the territory of the embolized vessels and therefore is frequently asymmetric. The gray matter is generally more severely affected than the white matter. The lesion margins are generally well delineated from normal tissue. Infarcted areas are usually ischemic but sometimes can also have a hemorrhagic component (2, 12).

### Acute non-compressive nucleus pulposus extrusion

Macroscopic examination of the vertebral canal allows identification of the gelatinous nucleus pulposus (generally on the right or left hand side of the vertebral canal) and the rent in the dorsal anulus fibrosus of the affected intervertebral disk (14). Mild epidural hemorrhage can sometimes be identified. Focal spinal cord contusion can generally be identified on external examination of the spinal cord segment overlying the affected intervertebral disk. On transverse section, spinal cord malacia with or without concurrent hemorrhage can be recognized (14). Microscopic changes are consistent with malacia of both white and gray matter with loss of normal architecture. Around the necrotic tissue, there are areas of degenerating white matter, diffuse microgliosis, and proliferating capillaries (14).

### Intradural/intramedullary intervertebral disc extrusion

Macroscopically, the spinal cord overlying the affected intervertebral disk may appear swollen, malacic, and hemorrhagic (24, 27). A rent can be identified in the dorsal anulus fibrosus of the affected intervertebral disk (23). The dura mater is lacerated dorsally to the affected intervertebral disk. Subdural hemorrhage may also be identified (24). Extension of the dural tear generally allows visualization of the intervertebral disk material when this has extruded in an intradural-extramedullary location. Myelotomy may be required to identify the intervertebral disk material when this has extruded within the spinal cord parenchyma. Histological examination of the affected spinal cord segments reveals malacia and hemorrhage of the white and gray matter with loss of normal architecture (24). Fragments of fibrocartilage can be identified within the spinal cord parenchyma (in dogs with intramedullary extrusions) or in an intradural-extramedullary location (23, 24, 27, 35). The fibrocartilage can either appear normal microscopically or have degenerated chondrocytes and focal mineralization (24, 28, 33).

## Signalment

Any breed of dog and cat of any gender can be affected by FCEM, ANNPE, or IIVDE. FCEM can occur in both adult and immature animals, whereas ANNPE or IIVDE have been reported only in animals older than 1 year.

### Fibrocartilaginous embolic myelopathy

Fibrocartilaginous embolic myelopathy has been reported most commonly in large and giant breed dogs of non-chondrodystrophic breeds. However, it has been described also in small breed dogs (particularly miniature schnauzers) (1, 3, 4, 6, 44). The male to female ratio in dogs ranges from 1:1 to approximately 2.5:1 in different studies. The age at diagnosis in dogs ranges from 2 months to 13 years and 5 months, with a median of 4–6 years in the majority of studies (13). In cats reported to date with FCEM or other causes of ischemic myelopathy, the domestic short hair is the most represented breed, the male to female ratio is 1.3:1, and the age at diagnosis ranges from 6 months to 17 years (median, 10 years) (10, 13).

### Acute non-compressive nucleus pulposus extrusion

Acute non-compressive nucleus pulposus extrusion has been reported in various canine breeds (predominantly non-chondrodystrophic) and in a few adult cats (14–22). Median age at diagnosis in dogs has been reported as 7 years (range, 2–11 years) and the reported male to female ratios have been 1.6:1 (18) and 2:1 (19). In the study including dogs with compressive and non-compressive myelopathy due to IVDE following trauma to the spinal region, dogs with spinal cord compression were significantly older and more likely to be chondrodystrophic and have evidence of generalized intervertebral disk degeneration, compared with dogs without spinal cord compression (20).

### Intradural/intramedullary intervertebral disc extrusion

Intradural/intramedullary intervertebral disk extrusion has been reported in various canine breeds and in two adult cats (23–37). By combining data of case reports and series 26 dogs with IIVDE have been identified. All 26 dogs were adults and the majority were middle to old aged (age range 1–14 years, median 9 years). There was no apparent gender predilection.

## Clinical Presentation

### Onset and inciting causes

The clinical presentation of dogs with FCEM, ANNPE, or IIVDE is typically characterized by peracute (<6 h) onset of neurological dysfunction referable to the site and extent of the spinal cord injury. Onset of neurological dysfunction during physical activity, such as running, jumping, or playing, is commonly reported at the onset of ANNPE, IIVDE, and FCEM (6, 18, 21, 25, 26, 31, 39). A history of blunt trauma to the spine at onset of neurological signs has been reported in dogs with ANNPE and IIVDE (18, 20, 24–26, 30). Vocalization, such as a yelp, scream, or cry as if in pain, is frequently observed at onset of neurological signs. Rarely for ANNPE and IIVDE and occasionally for FCEM, no apparent triggering event is identified (6, 22, 34, 35).

### Lesion localization and distribution

Fibrocartilaginous embolic myelopathy can occur in any spinal cord segment. Frequency of lesion localization varies among studies as well as between dogs and cats (13). In canine studies in which the diagnosis of FCEM has been confirmed histologically post-mortem, the C6–T2 and L4–S3 spinal cord segments are the most frequently affected (1, 3). Whereas when the diagnosis of FCEM is reached ante mortem the T3–L3 and L4–S3 spinal cord segments are the most commonly affected, followed by C6–T2 and lastly by C1–C5 (13).

Acute non-compressive nucleus pulposus extrusion and IIVDE have been reported most commonly in the T3–L3 spinal cord segments and particularly in the intervertebral disk spaces between T12 and L2 in dogs (18, 19, 23–27, 33, 34, 36, 37). The high frequency of occurrence of ANNPE and IIVDE at the junction between the mobile lumbar segment and the comparatively static thoracic segment of the vertebral column is likely attributable to the strong biomechanical forces that act at this junction, particularly during vigorous exercise or trauma. Changes in the orientation of the vertebral articular processes in the thoracolumbar region may also contribute to the increased susceptibility of this region (45).

Lateralization of neurological dysfunction has been reported as 53–87% in various canine studies on FCEM (1, 3, 5, 6, 9), 62–65% in dogs with ANNPE (18, 19), and 39% of dogs with IIVDE (by combining information of case reports in which information on neurological examination was available) (23–36). Generally, lateralization of neurological signs is marked in dogs with FCEM and ANNPE (e.g., severe paresis or plegia of the limb or limbs on one side of the body, and minimal neurological deficits in the contralateral limb or limbs) (Videos S1 and S2 in Supplementary Material). Conversely, dogs reported with IIVDE are severely paraparetic or paraplegic and the lateralization of signs consists of small differences in motor function or nociception between the two pelvic limbs (25, 27, 28, 30, 32, 34, 35).

In cats, FCEM has been reported to occur more frequently in the cervical spinal cord than in other locations (10, 13). In the largest feline study on ischemic myelopathy, lateralization of neurological signs occurred in 58% of cats (10). ANNPE has been reported in three cats at C2–C3, C3–C4, and L5–6, respectively (15, 21, 22). IIVDE has been reported at L4–L5 and L5–L6, respectively, in two domestic short hair cats (29, 46). Lateralization of neurological dysfunction was reported in these two cats with IIVDE as well as in the three cats with ANNPE.

### Spinal hyperalgesia

Spinal hyperalgesia on clinical examination performed within a few days of onset of neurological dysfunction is uncommon in dogs with FCEM (6) and has been reported in 21–57% of dogs with ANNPE (18, 19), and 5 (28%) of 18 dogs with IIVDE, in which information on neurological examination was available (23–36). Detection of spinal hyperalgesia is influenced by timing of clinical examination after onset of neurological dysfunction, the personality of the animal and administration of anti-inflammatory and analgesic medications (13).

## Imaging Findings

Magnetic resonance imaging is the diagnostic imaging modality of choice in supporting the ante mortem diagnosis of FCEM and ANNPE and can help to identify IIVDE (4, 6, 17, 18, 28, 29). Computed tomographic myelography can help in the diagnosis of IIVDE particularly when the intervertebral disk is in an intradural-extramedullary location (37).

### MRI findings-FCEM

**Figure 1:**
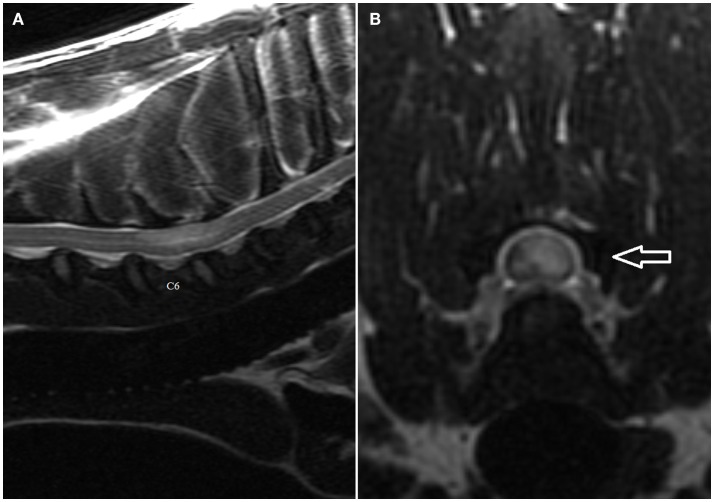
**(A,B) Sagittal (A) and transverse (B) T2-weighted magnetic resonance images of the cervical spine of the English Bull Terrier shown in Video S1 in Supplementary Material**. Note the intramedullary hyperintensity located above the C6 and the cranial half of C7 vertebral bodies. The spinal cord is swollen (Figure 2A). Note left-sided spinal cord intramedullary hyperintensity (arrow). There are no signal changes or extraneous material in the epidural space (Figure 2B).

The MRI features suggestive of FCEM include a focal, relatively sharply demarcated intramedullary, and often lateralized lesion (edematous infarcted tissue), predominantly involving the gray matter that is hyperintense to normal gray matter on T2-weighted (Figures 1A,B) and FLAIR images and iso- or hypointense to normal gray matter on T1-weighted images. Post-contrast T1-weighted images may show mild and heterogeneous enhancement of the affected area, generally on the fifth to seventh day of disease (4, 6). The length of the intramedullary hyperintensity on T2-weighted images is generally longer than one vertebral length (6). MRI performed 24–72 h after onset of neurological signs may reveal no intraparenchymal signal intensity changes in dogs with FCEM (6). Repeated MRI 72 or more hours after onset of neurological dysfunction may allow identification of the spinal cord intramedullary changes (6). Diffusion-weighted MRI has been shown to increase the sensitivity for diagnosis of spinal cord infarction in the early stages of the disorder (47, 48). In research dogs with spinal cord ischemia induced by embolization of the spinal branches of intercostal arteries, diffusion-weighted imaging with a 1.5-T MRI showed slight hyperintensity within 1 h postembolization in all six examined dogs (49). Clinical application of diffusion-weighted imaging is technically challenging due to its sensitivity to susceptibility artifacts of bone and motion artifacts of CSF. Using a multi-shot technique improves signal to noise ratio and decreases sensitivity to off-resonance effects (49).

### MRI findings-ANNPE

**Figure 2:**
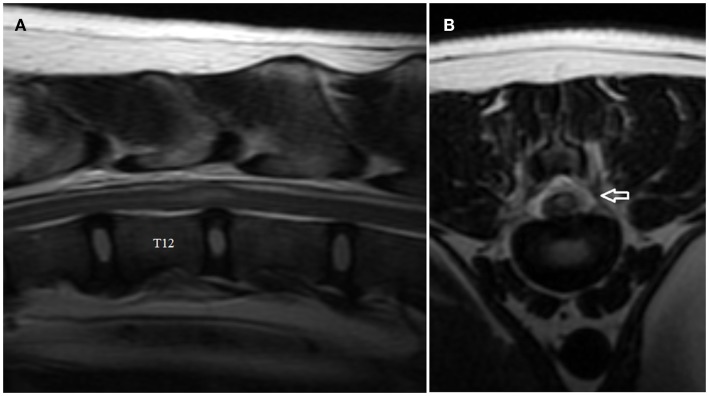
**Sagittal (A) and transverse (B) T2-weighted magnetic resonance images of the thoracolumbar spine of the boxer shown in Video S2 in Supplementary Material**. Note the intramedullary hyperintensity located predominantly above the T12–T13 intervertebral disk space and the decreased size of nucleus pulposus **(A)**. Note the signal change (arrow) in the left epidural space above the affected intervertebral disk and the left-sided spinal cord intramedullary hyperintensity **(B)**.

The MRI features of ANNPE include a focal area of hyperintensity on T2-weighted images within the spinal cord overlying an intervertebral disk space, with absent or minimal spinal cord compression (Figure 2A) (17). The area of intramedullary hyperintensity on T2-weighted images is often lateralized and it is generally less than one vertebral length (Figures 2A,B) (17, 18). The region of spinal cord that corresponds to the focal hyperintensity on T2-weighted images is most commonly isointense on T1-weighted images and does not have evidence of enhancement on T1-weighted images obtained after administration of contrast agent. However, hypointensity on T1-weighted images and mild heterogenous enhancement on post-contrast T1-weighted images rarely occur (17, 18). There is decreased size and signal intensity of the affected nucleus pulposus on T2-weighted images and the intervertebral disk space is often narrowed (17, 18). Extraneous material or signal change may be evident in the epidural space dorsal to the affected intervertebral disk (Figure 2B).

### MRI and computed tomographic myelography findings–IIVDE

**Figure 3:**
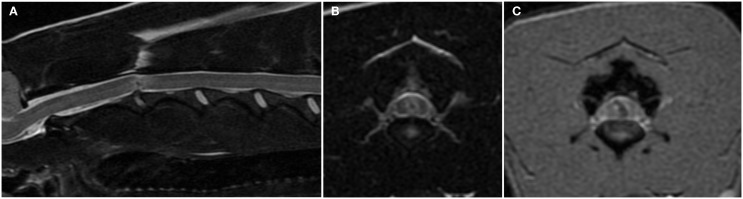
Sagittal (A) and transverse (B) T2-weighted and transverse T2*-weighted gradient recall echo (C) magnetic resonance images of the cervical spine of a 6-year-old female whippet with peracute onset non-ambulatory tetraparesis after running into a wall. The C2–C3 intervertebral disk space is narrowed and the nucleus pulposus is decreased in size and signal intensity **(A)**. The overlying spinal cord has intramedullary signal changes characterized by a focal hypointensity surrounded by ill-defined hyperintensity on T2-weighted images **(A,B)** and a curved linear hypointensity on T2*-weighted gradient recall echo images **(C)**. There are no signal changes or extraneous material in the epidural space.

In dogs with IIVDE resulting in intramedullary intervertebral disk penetration MRI allows visualization of signal changes within the affected intervertebral disk space and overlying spinal cord parenchyma (28, 29, 36, 46). The intervertebral disk space is generally narrowed and the nucleus pulposus has reduced signal intensity on T2-weighted images. The spinal cord overlying the affected intervertebral disk is swollen. The intramedullary signal changes include an hyperintensity on T2-weighted images and within it, one or more small focal areas of hypointensity on T2-weighted, T1-weighted, and T2*-weighted gradient recall echo images (Figures 3A,B) (28, 29, 36, 46). A linear tract (predominantly hyperintense on T2-weighted images, iso to hypointense on T1-weighted images and hypointense on T2*-weighted gradient recall echo images) extending from the intervertebral disk into the spinal cord parenchyma has been reported in two dogs and two cats undergoing high-field MRI and has been suggested to be specific for intramedullary IVDEs (28, 29, 36, 46). These intramedullary signal changes are most likely consistent with a mixture of extruded intervertebral disk material, edema, malacia, and hemorrhage. The appearance of hemorrhage on MRI is variable and depends upon numerous factors including the time hemorrhage occurred relative to the time of imaging, oxygen tension, size, and location of parenchymal hemorrhage, presence of hematoma, magnetic field strength, and pulse sequence (28, 29). T2*-weighted gradient recall echo images can be particularly helpful in identifying the intramedullary hemorrhage associated with the tract of the intramedullary extruded disk material (Figure 3C) (29, 46). Mild enhancement of the tissue adjacent to the tract can be observed on T1-weighted images following intravenous injection of paramagnetic contrast medium (29, 36, 46).

Results of a recent case series suggest that computed tomographic myelography may be more sensitive for diagnosing thoracolumbar IIVDE than low-field MRI, particularly when the intervertebral disk is in an intradural-extramedullary location (37). Computed tomographic myelography shows focal accumulation of iodinated contrast within the subarachnoid space and/or spinal cord parenchyma (30, 33, 35, 37) and in dogs with intradural-extramedullary IVDE, it can also allow identification of intervertebral disk material in the dilated subarachnoid space (35, 37). CT allows identification of calcified intervertebral disk material and differentiating it from hemorrhage as calcified tissue has higher Hounsfield unit than hemorrhage. Extradural leakage of iodinated contrast medium, suggestive of a dural tear, can sometimes be observed on computed tomographic myelography or myelography (25, 30). Traction of the cervical spine has been reported to help to identify a dural tear by allowing penetration of myelographic contrast medium through a defect in the dura mater and annulus fibrosus in dogs with cervical IIVDE (32).

## Treatment

Treatment of FCEM and ANNPE is conservative and includes nursing care and physical rehabilitation (18, 19). Anti-inflammatory and analgesic medications are administered to dogs with spinal hyperalgesia. Restricted physical activity for 4–6 weeks is often recommended in dogs with ANNPE and IIVDE to minimize the risk of further IVDE through the tear in the annulus fibrosus. Dogs reported with IIVDE have undergone either conservative treatment or surgical removal of the intervertebral disk material extruded intradurally and/or intramedullary (23–37). In some of the dogs reported with IIVDE, surgery was performed as exploratory procedure as the diagnosis was unclear following myelography or cross-sectional imaging (26–28, 31). Information in the veterinary literature is currently insufficient to provide evidence-based guidelines on whether and when surgery is indicated in dogs and cats with IIVDE. Advances in diagnostic imaging should help not only to diagnose IIVDE but also to provide criteria that could assist in treatment selection by differentiating compressive and non-compressive IIVDE. Estimation of the volume of intradural/extramedullary or intramedullary extruded intervertebral disk material and associated degree of spinal cord compression should help to select which patients would benefit of surgical decompression.

Dogs with a history of trauma should be thoroughly assessed for concurrent injuries and their blood pressure should be monitored and maintained within normal limits to ensure adequate perfusion of the damaged spinal parenchyma. Future advances in neuroprotective treatment for canine and feline spinal cord injury could help mitigate the damage to the spinal cord parenchyma following ischemic and contusive injuries (39).

## Outcome, Prognostic Factors, and Recovery Times

Although outcome definitions slightly differ between studies, a successful outcome generally refers to complete or partial recovery of neurological function, which allows performing daily activities as a functional pet (e.g., rising and walking unassisted, moving around the house, eating, drinking, going outside for walks and playing, being urinary and fecally continent) without extra care from the owner. Outcome has generally been defined as unsuccessful when the dog had residual severe neurological dysfunction, with or without episodic or persistent urinary or fecal incontinence, or was euthanatized because of a lack of recovery.

### Nociception

Loss of nociception has been reported as a negative prognostic factor for recovery in animals with spinal cord injury. Of 43 dogs with FCEM resulting in para or tetra plegia and loss of nociception, 42 dogs have been euthanized generally within 1 week of disease onset and 1 only has been reported to recover nociception and ambulatory status (1, 3, 5, 7). There is limited information on outcome of dogs with ANNPE or IIVDE and loss of nociception. Of 10 dogs with ANNPE resulting in paraplegia and loss of nociception in the pelvic limbs and tail, 5 were euthanized within 1 week of disease onset and 5 (with thoracolumbar ANNPE) recovered nociception and ambulatory status, however, had persistent neurological dysfunction including partial fecal and urinary incontinence (18, 19). Of three dogs with thoracolumbar IIVDE, paraplegia, and no nociception, one was euthanized soon after myelographic diagnosis, one recovered to a functional pet status following conservative management, and one recovered nociception and ambulatory status following surgery, however, had persistent neurological dysfunction including partial fecal and urinary incontinence (25, 36). Incomplete recovery with persistence of partial fecal and urinary incontinence has been reported also in dogs with thoracolumbar ANNPE (12 dogs) and IIVDE (1 dog), resulting in paraplegia with preserved nociception. These dogs had recovered motor function and unassisted ambulation (18, 19, 26). The fecal incontinence was characterized by the inability to control the urge to defecate for as long as the dogs could before ANNPE or IIVDE occurred and it was not perceived as a major problem by the owners, similar to what has been reported for dogs with severe compressive and contusive injuries to the thoracolumbar spinal cord (50).

### Degree of neurological dysfunction (neurological score)

Two studies have identified associations of outcome with degree of neurological dysfunction at initial examination quantified using a neurological score from 1 (clinically normal) to 5 (para or tetraplegia and loss of nociception) (7, 18). The median neurological score at presentation was 3 (non-ambulatory para or tetraparesis with or without concurrent mono or hemiplegia) in dogs with FCEM and 3.5 in dogs with ANNPE (7, 18). All dogs with a neurological score of 5 had an unsuccessful outcome. Of dogs with a neurological score of 4 (para or tetra plegia with preserved nociception), 6/20 (30%) dogs with FCEM and 6/13 (46%) dogs with ANNPE had an unsuccessful outcome. With the exception of one dog with FCEM and non-ambulatory tetra paresis, all dogs with FCEM or ANNPE and ambulatory or non-ambulatory para or tetra paresis (neurological score of 2 and 3, respectively) had a successful outcome. Involvement of an intumescence was not associated with outcome in both dogs with FCEM and ANNPE.

In a study comparing dogs with traumatic IVDE resulting in either compressive (9 dogs) or non-compressive (22 dogs) myelopathy, body weight, initial neurological score, outcome neurological score, and duration of hospitalization did not differ significantly between dogs with and without spinal cord compression (20). Of the nine dogs with spinal cord compression, two were euthanized immediately after the MRI, five underwent surgery and two dogs were managed with conservatively. Median initial neurological score was 3 (non-ambulatory para or tetraparesis) and median outcome neurological score (at the time of discharge or recheck 2–4 weeks after discharge) was 1 (spinal hyperesthesia only). Of the 22 dogs with non-compressive traumatic IVDE, 1 dog was lost to follow-up, 2 were euthanized immediately after MRI and the remaining 19 dogs had a median outcome neurological grade of 2 (ambulatory para or tetraparesis) at the time of discharge or recheck 2–4 weeks after discharge. The median neurological grade at initial presentation was 4 (tetra or paraplegia with nociception) (20).

### MRI findings

Associations between outcome and extent of the intramedullary hyperintensity on sagittal and transverse T2-weighted MR images has been reported in dogs with FCEM and ANNPE (7, 18). Cut-off values of measurements of extent of the intramedullary hyperintensity on sagittal and transverse T2-weighted MR images to maximize sensitivity when used to predict an unsuccessful outcome differ between dogs with FCEM and ANNPE (Table 1) (7, 18). In addition, in dogs with ANNPE detection of intramedullary hypointensity on gradient recall echo was associated with an unsuccessful outcome and multivariate analysis suggested that maximal cross-sectional area of the intramedullary hyperintensity on transverse T2-weighted MR images (expressed as a percentage of the cross-sectional area of the spinal cord at the same level) was the most useful MRI variable to predict outcome. The maximal cross-sectional area of the intramedullary hyperintensity on transverse T2-weighted MR images was also associated with time to recovery of unassisted ambulation in dogs with ANNPE, whereas no predictors of recovery times were identified in dogs with FCEM (7, 18).

**Table 1:** **Suggested cut-off values for lesion length to vertebral length ratio and percentage cross-sectional area of the intramedullary hyperintensity on T2-weighted magnetic resonance images to predict an unsuccessful outcome in dogs with fibrocartilaginous embolic myelopathy and acute non-compressive nucleus pulposus extrusion (6, 18)**.

	LL:VL	Sensitivity (%)	Specificity (%)	PCSAL (%)	Sensitivity (%)	Specificity (%)
FCEM	>2.0	100	62	≥67	100	52
ANNPE	>1.28	57	82	≥90	86	96

*FCEM, fibrocartilaginous embolic myelopathy; ANNPE, acute non-compressive nucleus pulposus extrusion; LL:VL, the ratio between the length of the intramedullary hyperintensity on mid-sagittal T2-weighted images and the length of either the C6 (for a cervical lesion) or L2 (for a thoracolumbar lesion) vertebral body; PCSAL, the maximal cross-sectional area of the lesion (largest region of intramedullary hyperintensity on transverse T2-weighted images) expressed as a percentage of the cross-sectional area of the spinal cord at the same level*.

### Recovery times

In dogs with FCEM, time intervals between onset of neurological signs and recovery of voluntary motor activity, unassisted ambulation, and maximal recovery have been reported as 6 days (range, 2.5–15 days), 11 days (range, 4–136 days), and 3.75 months (range, 1–12 months), respectively (7).

Time to recovery of unassisted ambulation in cats with FCEM has been reported to range from 2 to 27 days after diagnosis of FCEM (8–11, 13).

In dogs with ANNPE, time intervals between onset of neurological signs and recovery of nociception, voluntary motor activity, and unassisted ambulation have been reported as 6 days (range, 4–7 days), 6 days (range, 3–26 days), and 16.5 days (range, 2–93 days), respectively (18).

Information on recovery times in cats with ANNPE and in dogs and cats with IIVDE is limited.

## Conclusion

Differentiating between FCEM, ANNPE, and IIVDE can be challenging antemortem. Certain clinical and diagnostic imaging features can help discriminate between these conditions. Outcome is generally successful in dogs with residual motor function regardless of ambulatory status. Dogs with paraplegia and preserved nociception generally recover the ability to stand up and walk, however, partial fecal and urinary incontinence can persist. Dogs with loss of nociception rarely recover to a functional pet status. Disease-specific MRI variables may be particularly relevant to predict outcome in para or tetra-plegic dogs with or without nociception. To date, no study has directly compared outcomes in animals with FCEM, ANNPE, and IIVDE and therefore it is unknown if this differs significantly between these conditions.

## Conflict of Interest Statement

The author declares that the research was conducted in the absence of any commercial or financial relationships that could be construed as a potential conflict of interest.

## Supplementary Material

The Supplementary Material for this article can be found online at http://journal.frontiersin.org/article/10.3389/fvets.2015.00024

Video S1 **Nine-year-old, female spayed, English Bull Terrier with peracute onset of left-sided hemiparesis while playing in the garden 24 h before presentation**. Note the marked lateralization of neurological dysfunction.

Video S2 **Three-year five-month-old, male, Boxer with peracute onset of severe paresis in the left pelvic limb and mild paresis in the right pelvic limb while playing in the garden 7 h before presentation**. Note the marked lateralization of neurologic dysfunction. The decrease in the flexor withdrawal reflex in the left pelvic limb was transient and attributed to spinal shock at the time of presentation. Note the absence of the cutaneous trunci relfex on the left caudally to the dermatome of T13.

## References

[1] Cauzinille L Kornegay JN. Fibrocartilaginous embolism of the spinal cord in dogs: review of 36 histologically confirmed cases and retrospective study of 26 suspected cases. J Vet Intern Med (1996) 10:241–5. 10.1111/j.1939-1676.1996.tb02056.x 8819049

[2] Cauzinille L Fibrocartilaginous embolism in dogs. Vet Clin North Am Small Anim Pract (2000) 30:155–67. 10.1016/S0195-5616(00)50007-2 10680213

[3] Gandini G Cizinauskas S Lang J Fatzer R Jaggy A. Fibrocartilaginous embolism in 75 dogs: clinical findings and factors influencing the recovery rate. J Small Anim Pract (2003) 44:76–80. 10.1111/j.1748-5827.2003.tb00124.x 12622472

[4] Abramson CJ Garosi L Platt SR Dennis R McConnell JF. Magnetic resonance imaging appearance of suspected ischemic myelopathy in dogs. Vet Radiol Ultrasound (2005) 46:225–9. 10.1111/j.1740-8261.2005.00037.x 16050280

[5] Dunié-Mérigot A Huneault L Parent J. L’embolie fibrocartilagineuse chez le chien: une étude retrospective. Can Vet J (2007) 48:63–8. 17310624 PMC2831623

[6] De Risio L Adams V Dennis R McConnell F Platt S. Magnetic resonance imaging findings and clinical associations in 52 dogs with suspected ischemic myelopathy. J Vet Intern Med (2007) 21:1290–8. 10.1111/j.1939-1676.2007.tb01951.x 18196739

[7] De Risio L Adams V Dennis R McConnell F Platt SR. Association of clinical and magnetic resonance imaging findings with outcome in dogs suspected to have ischemic myelopathy: 50 cases (2000-2006). J Am Vet Med Assoc (2008) 233:129–35. 10.2460/javma.233.1.129 18593322

[8] Mikszewski JS Van Winkle TJ Troxel MT. Fibrocartilaginous embolic myelopathy in five cats. J Am Anim Hosp Assoc (2006) 42:226–33. 10.5326/0420226 16611936

[9] Nakamoto Y Ozawa T Mashita T Mitsuda M Katakabe K Nakaichi M. Clinical outcomes of suspected ischemic myelopathy in cats. J Vet Med Sci (2010) 72:1657–60. 10.1292/jvms.10-0121 20710125

[10] Theobald A Volk HA Dennis R De Risio L. Clinical outcome in 19 cats with clinical and magnetic resonance imaging diagnosis of ischaemic myelopathy (2000-2011). J Feline Med Surg (2013) 15:132–41. 10.1177/1098612X12463927 23048075 PMC10816650

[11] Simpson KM De Risio L Theobald A Garosi L Lowrie M. Feline ischaemic myelopathy with a predilection for the cranial cervical spinal cord in older cats. J Feline Med Surg (2014) 16:1001–6. 10.1177/1098612X14522050 24509256 PMC11104088

[12] De Risio L Platt SR. Fibrocartilaginous embolic myelopathy in small animals. Vet Clin North Am Small Anim Pract (2010) 40:859–69. 10.1016/j.cvsm.2010.05.003 20732595

[13] De Risio L What is fibrocartlagenous embolism and is it related to IVDD? In: Fingeroth J Thomas W, editors. Advances in Intervertebral Disc Disease in Dogs and Cats. Iowa:Wiley-Blackwell (2015). p. 75–88.

[14] Griffiths IR A syndrome produced by dorso-lateral “explosions” of the cervical intervertebral discs. Vet Rec (1970) 87:737–41. 10.1136/vr.87.24.737 5531244

[15] Lu D Lamb CR Wesselingh K Targett MP. Acute intervertebral disc extrusion in a cat: clinical and MRI findings. J Feline Med Surg (2000) 4:65–8. 10.1053/jfms.2001.0150 11869056 PMC10829150

[16] Bagley RS Spinal cord enigmas: fibrocartilagenous emboli, arachnoid cyst, and others. Proceedings of 21st Annual American College of Veterinary Internal Medicine Forum (2003). p. 10–1.

[17] Chang Y Dennis R Platt SR Penderis J Magnetic resonance imaging features of traumatic intervertebral disc extrusion in dogs. Vet Rec (2007) 160:795–9. 10.1136/vr.160.23.795 17558027

[18] De Risio L Adams V Dennis R McConnell FJ. Association of clinical and magnetic resonance imaging findings with outcome in dogs with presumptive acute noncompressive nucleus pulposus extrusion: 42 cases (2000-2007). J Am Vet Med Assoc (2009) 234(4):495–504. 10.2460/javma.234.4.495 19222359

[19] McKee WM Downes CJ Pink JJ Gemmill TJ. Presumptive exercise-associated peracute thoracolumbar disc extrusion in 48 dogs. Vet Rec (2010) 166:523–8. 10.1136/vr.b4823 20418513

[20] Henke D Gorgas D Flegel T Vandevelde M Lang J Doherr MG Magnetic resonance imaging findings in dogs with traumatic intervertebral disk extrusion with or without spinal cord compression: 31 cases (2006-2010). J Am Vet Med Assoc (2013) 242:217–22. 10.2460/javma.242.2.217 23276099

[21] Sanders S Bagley RS Tucker RL Nelson NR Radiographic diagnosis: focal spinal cord malacia in a cat. Vet Radiol Ultrasound (1999) 40:122–5. 10.1111/j.1740-8261.1999.tb01895.x 10225521

[22] Chow K Beatty JA Voss K Barrs VR. Probable lumbar acute non-compressive nucleus pulposus extrusion in a cat with acute onset paraparesis. J Feline Med Surg (2012) 14:764–7. 10.1177/1098612X12450110 22661021 PMC11104097

[23] Montavon PM Weber U Guscetti F Sutter PF What is your diagnosis? Swelling of spinal cord associated with dural tear between segments T13 and L1. J Am Vet Med Assoc (1990) 196:783–4. 2307618

[24] Roush JK Douglass JP Hertzke D Kennedy GA Traumatic dural laceration in a racing greyhound. Vet Radiol Ultrasound (1992) 33:22–4. 10.1111/j.1740-8261.1992.tb01951.x

[25] Hay CW Muir P. Tearing of the dura mater in three dogs. Vet Rec (2000) 146:279–82. 10.1136/vr.146.10.279 10749041

[26] Yarrow TG Jeffery ND Dura mater laceration associated with acute paraplegia in three dogs. Vet Rec (2000) 146:138–9. 10.1136/vr.146.5.138 10706333

[27] Liptak JM Allan GS Krockenberger MB Davis PE Malik R Radiographic diagnosis: intramedullary extrusion of an intervertebral disk. Vet Radiol Ultrasound (2002) 43:272–4. 10.1111/j.1740-8261.2002.tb01002.x 12088323

[28] Sanders SG Bagley RS Gavin PR. Intramedullary spinal cord damage associated with intervertebral disk material in a dog. J Am Vet Med Assoc (2002) 221:1594–6. 10.2460/javma.2002.221.1594 12479331

[29] McConnell JF Garosi LS. Intramedullary intervertebral disk extrusion in a cat. Vet Radiol Ultrasound (2004) 45:327–30. 10.1111/j.1740-8261.2004.04062.x 15373259

[30] Packer RA Frank PM Chambers JN. Traumatic subarachnoid-pleural fistula in a dog. Vet Radiol Ultrasound (2004) 45:523–7. 10.1111/j.1740-8261.2004.04089.x 15605842

[31] Meola SD Swiderski JK Randall EK Kraft S Palmer RH What is your diagnosis? J Am Vet Med Assoc (2007) 230:1629–30. 10.2460/javma.230.11.1629 17542726

[32] McKee WM Downes CJ. Rupture of the dura mater in two dogs caused by the peracute extrusion of a cervical disc. Vet Rec (2008) 162:479–81. 10.1136/vr.162.15.479 18408196

[33] Kent M Holmes S Cohen E Sakals S Roach W Platt S Imaging diagnosis-CT myelography in a dog with intramedullary intervertebral disc herniation. Vet Radiol Ultrasound (2011) 52:185–7. 10.1111/j.1740-8261.2010.01755.x 21388471

[34] Poncelet L Heimann M Intradural lumbar disk herniation in a dog. Vet Rec (2011) 168:486 10.1136/vr.c6740 21527476

[35] Barnoon I Chai O Srugo I Peeri D Konstantin L Brenner O Spontaneous intradural disc herniation with focal distension of the subarachnoid space in a dog. Can Vet J (2012) 53:1191–4. 23633713 PMC3474575

[36] Kitagawa M Okada M Kanayama K Sakai T. Identification of ventrolateral intramedullary intervertebral disc herniation in a dog. J S Afr Vet Assoc (2012) 83:103. 10.4102/jsava.v83i1.103 23327141

[37] Tamura S Doi S Tamura Y Takahashi K Enomoto H Ozawa T Thoracolumbar intradural disc herniation in eight dogs: clinical, low-field magnetic resonance imaging, and computed tomographic myelography findings. Vet Radiol Ultrasound (2015) 56:160–7. 10.1111/vru.12213 25263808

[38] Hansen HJ A pathologic-anatomical study on disc degeneration in dog. Acta Orthop Scand (1952) 11:1–117. 10.3109/ort.1952.23.suppl-11.01 14923291

[39] Jeffery ND Levine JM Olby NJ Stein VM. Intervertebral disk degeneration in dogs: consequences, diagnosis, treatment, and future directions. J Vet Intern Med (2013) 27:1318–33. 10.1111/jvim.12183 24010573

[40] Funquist B Thoraco-lumbar disk protrusion with severe cord compression in the dog. I. Clinical and patho-anatomic observations with special reference to the rate of development of the symptoms of motor loss. Acta Vet Scand (1962) 3:256–74.

[41] Beltran E Dennis R Doyle V de Stefani A Holloway A De Risio L. Clinical and magnetic resonance imaging features of canine compressive cervical myelopathy with suspected hydrated nucleus pulposus extrusion. J Small Anim Pract (2012) 53:101–7. 10.1111/j.1748-5827.2011.01166.x 22250580

[42] Hamilton T Glass E Drobatz K Agnello KA. Severity of spinal cord dysfunction and pain associated with hydrated nucleus pulposus extrusion in dogs. Vet Comp Orthop Traumatol (2014) 4:313–8. 10.3415/VCOT-13-06-0076 24992451

[43] Manunta ML Evangelisti MA Bergknut N Grinwis GC Ballocco I Meij BP. Hydrated nucleus pulposus herniation in seven dogs. Vet J (2015) 203:342–4. 10.1016/j.tvjl.2014.12.027 25599897

[44] Hawthorne JC Wallace LJ Fenner WR Waters DJ. Fibrocartilaginous embolic myelopathy in miniature schnauzers. J Am Anim Hosp Assoc (2001) 37:374–83. 10.5326/15473317-37-4-374 11450839

[45] Breit S. Functional adaptations of facet geometry in the canine thoracolumbar and lumbar spine (Th10-L6). Ann Anat (2002) 184:379–85. 10.1016/S0940-9602(02)80059-0 12201048

[46] Hammond LJ Hecht S. Susceptibility artifacts on T2*-weighted magnetic resonance imaging of the canine and feline spine. Vet Radiol Ultrasound (2015) 56(4):398–406. 10.1111/vru.12245 25693447

[47] Thurnher MM Bammer R. Diffusion-weighted MR imaging (DWI) in spinal cord ischemia. Neuroradiology (2006) 48(11):795–801. 10.1007/s00234-006-0130-z 16977443

[48] Weidauer S Nichtweiß M Hattingen E Berkefeld J. Spinal cord ischemia: aetiology, clinical syndromes and imaging features. Neuroradiology (2015) 57(3):241–57. 10.1007/s00234-014-1464-6 25398656

[49] Zhang JS Huan Y Sun LJ Ge YL Zhang XX Chang YJ. Temporal evolution of spinal cord infarction in an in vivo experimental study of canine models characterized by diffusion-weighted imaging. J Magn Reson Imaging (2007) 26(4):848–54. 10.1002/jmri.21044 17896378

[50] Olby N Levine J Harris J Munana K Skeen T Sharp N. Long-term functional outcome of dogs with severe injuries of the thoracolumbar spinal cord: 87 cases (1996-2001). J Am Vet Med Assoc (2003) 222:762–9. 10.2460/javma.2003.222.762 12675299

